# Retrospective Clinical Study of a Freely Removable Implant-Supported Fixed Dental Prosthesis by a Microlocking System

**DOI:** 10.1155/2020/7929585

**Published:** 2020-11-03

**Authors:** Eun-Bin Bae, Won-Tak Cho, Hyun-Young Bae, So-Hyoun Lee, Tae-Hyung Kim, Jung-Bo Huh

**Affiliations:** ^1^Department of Prosthodontics, Dental Research Institute, Dental and Life Science Institute, BK21 PLUS Project, School of Dentistry, Pusan National University, Geumo-ro 20, 50612, Republic of Korea; ^2^Removable Prosthodontics, Division of Restorative Sciences, Herman Ostrow School of Dentistry, University of Southern California, 938 W 34th Str, 90007, USA

## Abstract

This retrospective clinical study was conducted to evaluate the clinical usefulness of a freely removable microlocking implant prosthesis (MLP) that was developed to overcome the problems with conventional implant prostheses. A total of 54 patients (male: 31, female: 23) and 100 implant prostheses were included. Patients were divided into three groups such as 6-12 months, 12-18 months, and 18-24 months according to the used period after implant prosthesis delivery, and the patients in each group were recalled for examinations of survival rate, marginal bone resorption, peri-implant soft tissue indices, and complications. The prosthetic complications were analysed by combining the recorded chart data during the periodic checks including the last call for this study. During a 2-year observation period, all the implants showed a 100% survival rate without clinical mobility and functional problems. There was no significant difference in marginal bone resorption, plaque index, and bleeding index over the observation period after implant prosthesis delivery. Probing depth of the 18-24 months group (1.5 ± 0.19 mm) was significantly lower than that of the 6-12 months group (*p* < 0.05). The main complication was abutment loosening (4%), followed by implant prosthesis fracture (2%) and food impaction (2%) which were recorded. Within the limits of the present study, the implant prostheses with MLP are considered to be an applicable and predictable treatment method.

## 1. Introduction

Recovery of missing teeth using a dental implant does not damage adjacent teeth and offers the patient aesthetic and functional advantages when compared to removable dentures. As the use of implants is gradually expanded due to these advantages, various types of implant systems have been developed, and the results of evaluating the clinical success of inserted implants have been reported continuously [1–4]. Retained types of implant-supported fixed dental prostheses (FDPs) can be typically divided into screw-retained and cement-retained types [5, 6]. The success rate of implant was not affected by both retained types of implant prosthesis [7], but both types had relative pros and cons and could affect the frequency of biological and mechanical complications [8, 9].

A screw-retained prosthesis facilitates removal, repairs, and hygiene maintenances of the implant prosthesis [10] and prevents complications such as peri-implantitis, edema, and ulcer due to residual cement around the abutment and implant prosthesis [11, 12]. On the other hand, a screw access hole in the occlusal table can interfere with the assignment of aesthetic and proper occlusal contact points, and mechanical complications such as screw loosening and fracture of prosthesis can occur [6, 9, 13]. In the case of cement-retained prosthesis, it is easy to achieve passive fit of prosthesis, there is no screw access hole in the occlusal table, and occlusion can be easily controlled [10, 14], but it is not easy to remove excessive cement around the prosthesis. Furthermore, it can promote peri-implant diseases such as peri-implant mucositis and peri-implantitis associated with residual cement [8, 10, 15]. To overcome these problems, a screw-and-cement-retained prosthesis (SCRP), which combines the advantages of screw-retained and cement-retained prostheses, was introduced [16]. However, such prostheses may also be restricted by an inappropriate screw access hole within the occlusal surface because of implant angular placement [17].

Recently, a novel implant prosthetic system (microlocking implant prosthetic system (MLP); EZ crown; Samwon DMP), which is a freely removable using zirconia ball and Ni-Ti spring, has been developed to compensate for the shortcomings of the existing implant prosthetic system [18, 19]. The MLP achieves aesthetics, occlusal stability, and passive suitability of prosthesis, and it can prevent the mechanical (e.g., screw loosening and fracture) and biological complications (e.g., peri-implantitis) because prostheses can be delivered without screws or cement [14, 20]. Several case studies have reported successful use of MLP [18, 19], but there were no studies reporting the objective efficacy and safety of MLP based on clinical outcomes. Until now, the previous assessments of correlation between implant-related complications and implant survival or success rates were limited to the screw-retained prosthesis or cement-retained prosthesis [12, 21].

The MLP investigated in this study consists of completely different components, which may lead to unreported prosthetic complications previously. So, long-term clinical observations are very important because these prosthetic changes can affect the implant success rate. Thus, the present study investigated the periodontal indices and complications in the cases using MLP through retrospective clinical examination and radiological analysis. The purpose of this study was to evaluate the clinical usefulness and complications of the new developed MLP.

## 2. Materials and Methods

### 2.1. Research Subjects

For the retrospective evaluation of the MLP, in 54 patients (male: 31, female: 23) who visited Pusan National University Dental Hospital from 2016 to 2019, 100 implant prostheses were evaluated after at least 6 months after implant prosthesis delivery (Tables 1 and 2). This study evaluated patients who were over 20 years old among the partially edentulous patients and who had no uncontrolled systemic disease. Patients who had difficulty in a regular follow-up for clinical evaluation after implant prosthesis delivery were excluded (IRB no. PNUDH-2017-035-MD).

### 2.2. Microlocking Implant Prosthesis (MLP)

**Figure 1:**
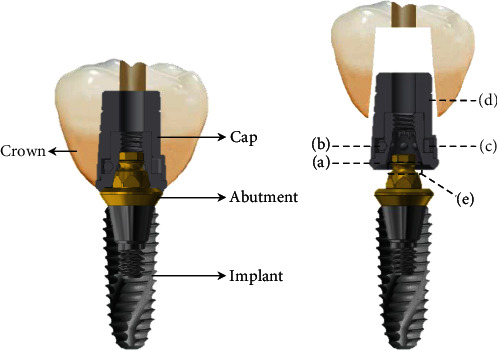
Detailed illustration of the abutment and cap making up the MLP: (a) body, (b) ball, (c) spring, (d) cap, and (e) retention groove of the abutment.

The MLP evaluated in this study consists of a fixture, abutment, and cap, and the cap consists of four subcomponents: body, ball, spring, and cap. The body has several grooved hexagonal receptacles that match the hexagonal structure of the abutment cylinder to prevent the rotation of the prosthesis. The main components of the balls are zirconium oxide (ZrO₂) and hafnium oxide (HfO₂), and balls are seated in the retention groove so that they are directly involved in the retention and prevent the rotation of the spring. The spring consists of nickel-titanium (Ni-Ti) shape memory alloy and is enveloping the outside of the zirconia balls. This structure maintains a constant stress value, and retentive components are restored even in large deformational distortion. So, the prosthesis can be attached and detached without deformation or loss of retention. The spring used in this study expands slightly when the cap is engaged with the abutment, and the cap can be easily positioned on the undercut of the retention groove and applies a constant external force to the ball after the prosthesis is combined with the implant (Figure 1) [18].

**Figure 2:**
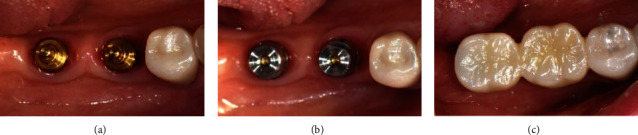
Prosthesis setting process of MLP: (a) properly selected abutments were connected to the fixtures; (b) caps were attached on the abutments; (c) fabricated zirconia crowns were cemented on the caps with resin cement.

Before making the MLP impression, the abutment was tightened to 35 Ncm according to the manufacturer's instruction (Figure 2(a)), and then, the cap was attached to the abutment using a dedicated tool (Figure 2(b)). Impressions obtained using silicone impression materials (Imprint II VPS Impression Material; 3 M ESPE) were scanned using a three-dimensional (3D) scanner (Trios 3; 3shape), and a zirconia crown was fabricated with a computer-aided design and computer-aided manufacturing (CAD-CAM) system (Exocad Dental CAD; Exocad GmbH, Trione Z; DIO). Prior to the final cementation of the crown, the crown was evaluated regarding the marginal fit, aesthetics, and occlusion in the oral cavity and was finally cemented to the cap using self-adhesive resin cement (G-CEM LinkAce; GC America). After removing the cemented crown and cap using a dedicated removal driver, the excessive cement was then cleaned and the crown margin area was polished. The crown cemented with a cap was inserted on the abutment again, and the access hole on the occlusal surface was filled with a flowable composite resin (Filtek Z350 XT; 3 M ESPE) (Figure 2(c)) [19].

### 2.3. Clinical Examination

**Figure 3:**
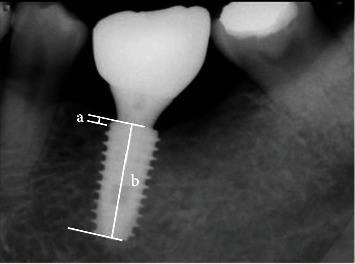
References used to measure actual marginal bone loss: (a) marginal bone level (distance from the implant platform to the top of the marginal bone) and (b) distance of the implant.

After the final implant prosthesis delivery, the patients were divided into three groups: (1) 6-12 months, (2) 12-18 months, and (3) 18-24 months, according to the used observation periods. The following items were evaluated with references to clinical examination and radiographs from implant placement to the final visit. The cumulative implant survival rates were assessed according to the criteria presented by Cochran et al. [22]. The evaluation criteria are as follows: (1) persistent or no recurrence of infection around the implant; (2) no persistent discomfort such as pain, foreign body sensation, and neurological abnormality; (3) no clinical mobility of the implant; and (4) no radiological transmission and rapid progression of bone loss around the implant. For analysing implant marginal bone resorption, radiographs were taken with a paralleling technique using a portable radiographic device (PORT-X II, Genoray). The radiographs taken at the last visit and at the final prosthesis delivery were compared to evaluate the peri-implant bone loss. The obtained images were accessed by using an image measurement program (i-Solution; IMT), and the mean and standard deviation were calculated after compensating for the amount of marginal bone resorption compared to the length of implant fixture (Figure 3) [23].

The probing depth was measured in parallel with implant length at four points around the implant (mesial, distal, buccal, and lingual) using a periodontal probe (Merrit-B; Hu-Friedy) at the final recall check, and the mean and standard deviation were calculated [24]. According to the criteria set by Mombelli et al. [25], the modified plaque index (mPI) measured the plaque attached to the implant surface at the final recall check, and scores from 0 to 3 were checked. The modified sulcus bleeding index (mBI) was measured using a periodontal probe (Merrit-B; Hu-Friedy) according to the criteria of Mombelli et al. [25] at the final recall check. The complications were investigated after implant prosthesis delivery, and the classified items of complications and their frequencies were recorded. Complications were examined for all problems found in the prostheses themselves and counted for all complications listed on the chart during the regular and final checks for this study. The same complications that occurred several times in one implant were counted repeatedly.

For the statistical analysis, one-way analysis of variance (ANOVA) with post hoc Tukey's test was performed to compare the marginal bone resorption between groups. In the results of probing depth, the Kruskal-Wallis (KW) test with the post hoc Mann-Whitney *U* test was used to confirm the significance between groups. The significant differences of the mPI and mBI were confirmed by the chi-square test. All the statistical processes were based on SPSS 25 (IBM) at a significance level of 5%.

## 3. Results

### 3.1. Cumulative Implant Survival Rate

Two-year cumulative survival rates of 100 implants with MLP were evaluated in 54 patients. All the implants showed no clinical mobility and functional problems, yielding a cumulative survival rate of 100% (Table 3).

### 3.2. Implant Marginal Bone Resorption

The mean and standard deviations of implant marginal bone resorption are shown in Table 4. At 6-12, 12-18, and 18-24 months after implant prosthesis delivery, the marginal bone resorption was 0.24 ± 0.47 mm, 0.21 ± 0.54 mm, and 0.38 ± 0.34 mm, respectively. There was no significant difference among the three groups (*p* > 0.05).

### 3.3. Probing Depth

The mean and standard deviations of probing depth are shown in Table 5. The probing depths at 6-12, 12-18, and 18-24 months after implant prosthesis delivery were 2.12 ± 0.54 mm, 1.87 ± 0.49 mm, and 1.5 ± 0.19 mm, respectively. The 6-12 months group showed higher probing depth than the 18-24 months group (*p* < 0.05).

### 3.4. Modified Plaque Index (mPI) and Modified Sulcus Bleeding Index (mBI)

In all groups, the score of 0 was most frequently observed for both mPI and mBI, but there was no statistically significant difference among the three groups (*p* > 0.05; Table 6).

### 3.5. Prosthetic Complication

The total incidence of complications was most frequently observed at 12-18 months after implant prosthesis delivery. As the main complication, abutment loosening (1%) in the 6-12 months group, abutment loosening (2%) and food impaction (2%) in the 12-18 months group, and abutment loosening (1%) and implant prosthesis fracture (1%) in the 18-24 months group were most frequently observed (Table 7). In particular, abutment loosening was observed in all groups, and all of them were found in the posterior area. Other complications were not recorded except for abutment loosening, implant prosthesis fracture including zirconia crown fracture and connector fracture in cases of bridge prostheses, and food impaction caused by loosening of contact points with the proximal tooth.

## 4. Discussion

The screw-retained type of implant-supported FDPs, which are widely used in clinical practice, may cause screw loosening, screw fracture, or fracture of implant abutment [26]. Cement-retained types also have some limitations, such as infection and marginal bone resorption due to residual cement. In addition, it is reported that the crown margin can be located below the soft tissue margin for aesthetic reasons, in which case excessive cement removal may damage the soft tissue around the implant [27]. As a result, these cases can be susceptible to peri-implantitis. To overcome these shortcomings, the MLP has been recently developed and introduced. The use of MLP improves the problem of nonaesthetic elements and occlusal contact formation due to large screw holes. In addition, it is possible to freely attach and detach the prosthesis to abutment so that periodontitis caused by excess cement can be prevented. Adjusting the occlusion contact and removing the cement are easy in MLP using a zirconia ball and Ni-Ti spring because it does not affect the screw even though it is repeatedly attached and detached, unlike the conventional type [18–20].

Although it has short duration of observation, the two-year retrospective clinical study conducted to evaluate the clinical stability showed the cumulative implant survival rate of 100%. Based on these results, it seems that the change in the retained structure of the implant prosthesis does not significantly affect the implant survival rate within the initial 2 years. For marginal bone resorption, the previous study reported a screw-retained prosthesis (0.4 ± 0.3 mm) and cement-retained prosthesis (0.3 ± 0.6 mm) [28]. Marginal bone resorption of the screw-retained prosthesis (0.8 ± 0.8 mm) and cement-retained prosthesis (0.8 ± 0.4 mm) was reported in the clinical follow-up research of submerged and internal connection implants about four years [29, 30]. In the present study, the marginal bone resorption of the MLP was found to be insignificant compared to the previous studies [28–30], and this study also compared the implant soft tissue condition surrounding the MLP using several parameters to measure mPI and mBI. In the study comparing a cement-retained prosthesis with a screw-retained prosthesis, cement-retained prostheses generally showed greater plaque accumulation and bleeding than screw-retained prosthesis during the follow-up [31]. Whereas the periodontal indices of a cement-retained prosthesis remained to have a high value for three years of observation, the screw-retained prosthesis showed decreased plaque retention and stable bleeding levels after six months [31]. Similarly, MLP also improved over time with mPI and mBI scores. This suggests that the overall periodontal indices of the MLP during the observation period are better than those of the cement-retained prosthesis presented in the previous studies.

The main complications of the screw-retained prosthesis were screw loosening, abutment loosening, ceramic fracture, loss of composite resin in the screw access hole, and abutment fracture [5, 26, 32–35]. In the case of the cement-retained prosthesis, screw loosening, ceramic fracture, abutment fractures, and debonding of the cemented crown have been reported as the main complications [5, 26, 32–35]. In the present study, the abutment loosening was a major complication of MLP, and the implant prosthesis fracture and food impaction were found additionally. When comparing the incidence of abutment loosening with that in other implant systems, it was found that the incidence in MLP was slightly higher than that in other systems [36]. In the case of MLP, the abutment is made compatible with internal and submerged fixtures with a Morse taper from various companies. For this reason, in this study, the MLP was used for various kinds of fixtures manufactured by diverse companies. There may have been a slight difference in the connection part of fixture. Therefore, the higher abutment loosening frequency might be due to the microgap or incorrect joint between the connection part of fixture and the abutment. Besides, MLP is commercially available recently and is lacking a variety of abutments with heights and diameters in comparison with other systems yet. In general, the larger height of the abutment and the wider prosthetic table width make the smaller lateral or rotational micromovement of the prosthesis [37, 38]. Although it is our subjective opinion, abutment loosening, which was reported as a frequent complication in this study, may be caused by a small prosthetic table width in the abutment. Therefore, in the future, the development of abutments with prosthetic table widths of various diameters, as well as the development of one-piece implants in which the fixture and abutment are integrated, may be considered. The fracture of an implant prosthesis occurred when the prosthesis was made of zirconia, which may be due to failure to establish proper zirconia thickness, design problems, or defects in the zirconia block itself [39]. Therefore, implant prosthesis fracture is considered a common complication of dental prosthesis, independent of implant abutment [40]. Additionally, the food impaction may occur by the loosening of the contact between adjacent teeth. Meanwhile, Choi et al. [20] reported that problems with food collection between the abutment and prosthetics were observed in MLP. However, there is no evidence in this study that these problems affect periodontal tissue or marginal bone resorption. The previous study of the MLP reported that the load-bearing capacity of the MLP was not significantly different from that of other commercially available systems [18].

In this clinical study, no fracture or deformation of MLP components was observed and the structural stability was confirmed. However, this study is a retrospective study based on a chart review that recorded the clinical evaluation and radiographic evaluation. So, the factors that can be evaluated in the present study were limited. In addition, since the MLP system was a recently developed system, the amount of collected data was smaller and the follow-up period was shorter compared to the conventional types (e.g., screw retention and cement retention). Long-term clinical observations for more subjects should be conducted to verify these clinical findings of the present study. Moreover, other complications not found in this study can be identified through further studies, and it may help to find the correlation with the pathology of soft and hard tissues around the implants [31].

## 5. Conclusions

The present study attempted to confirm the clinical usefulness of the MLP through a 2-year follow-up of the clinical progress of the implant prosthesis. All implants had no clinical and functional problems, but abutment loosening was observed in four of the 100 implant prostheses, and further research should be conducted to get to the bottom of the problem. Within the limits of the present study, the implant prostheses with MLP are considered to be an applicable and predictable treatment method. However, more samples and long-term clinical studies should be conducted to establish reliable evidence.

## Figures and Tables

**Table 1:** Distribution of implant placement.

Location	Anterior	Premolars	Molars	Total (*n*)
Maxilla	1	17	10	28
Mandible	2	18	52	72

**Table 2:** Distribution of implants according to the implant length and diameter.

Length (mm)	Diameter (mm)					Total (*n*)
	3.5	4.0	4.5	4.8	5.0	
7.0					2	2
8.0		2	11		4	17
8.5		3		1	4	8
10.0	1	23	12	1	16	53
11.5		3	3		7	13
12.0	1	4	1			6
13.0			1			1
Total (*n*)	2	35	28	2	33	100

**Table 3:** Cumulative survival rate of implants.

	Implants	Failed implants	CSR (%)
6-12 months	41	—	100
12-18 months	40	—	100
18-24 months	19	—	100

CSR = cumulative survival rate of implants.

**Table 4:** The average value of marginal bone resorption.

	Observation period	Mean ± SD	*p* value
Marginal bone resorption (mm)	6-12 months	0.24 ± 0.47	0.452
	12-18 months	0.21 ± 0.54	
	18-24 months	0.38 ± 0.34	

SD = standard deviation.

**Table 5:** The average value of probing depth.

	Observation period	Mean ± SD	*p* value
Probing depth (mm)	6-12 months	2.12 ± 0.54^a^	0.035^∗^
	12-18 months	1.87 ± 0.49	
	18-24 months	1.5 ± 0.19^a^	

^∗^ *p* < 0.05. ^a^Statistically significant difference (*p* = 0.02).

**Table 6:** Modified plaque index (mPI) and modified sulcus bleeding index (mBI).

	Score	Occurrence rate (%)			*p* value
		6-12 months	12-18 months	18-24 months	
mPI	0	83.3	85.7	100.0	0.78
	1	13.9	14.3	—	
	2	2.7	—	—	
	3	—	—	—	

mBI	0	72.2	81	100.0	0.60
	1	16.7	14.3	—	
	2	11.1	4.8	—	
	3	—	—	—	

mPI = modified plaque index; score 0: no detection of plaque; score 1: plaque only recognized by running a probe across the smooth marginal surface of the implant; score 2: plaque can be seen by the naked eye; score 3: abundance of soft matter. mBI = modified sulcus bleeding index; score 0: no bleeding when a periodontal probe is passed along the gingival margin adjacent to the implant; score 1: isolated bleeding spots visible; score 2: blood forms a confluent red line on the margin; score 3: heavy or profuse bleeding.

**Table 7:** The detected clinical prosthetic complications according to the observation period.

Complications	Groups according to the observation period			Total (*n*)
	6-12 months	12-18 months	18-24 months	
Abutment loosening	1	2	1	4
Implant prosthesis fracture	—	1	1	2
Food impaction	—	2	—	2
Total (*n*)	1	5	2	8

## Data Availability

The data used to support the findings of this study are available from the corresponding author upon request.
